# Research progress of NAC transcription factors in woody plants

**DOI:** 10.3389/fpls.2025.1592898

**Published:** 2025-06-04

**Authors:** Huan Zhang, Jia Zhao, Tongxing Zhang, Guiying Wang, Zhixiao Han, Yuemei Meng, Jingran Bi, Yachao Ren, Minsheng Yang

**Affiliations:** ^1^ Institute of Forest Biotechnology, Forestry College, Hebei Agricultural University, Baoding, China; ^2^ Hebei Key Laboratory for Tree Genetic Resources and Forest Protection, Baoding, China; ^3^ Flower Research Institute, Langfang Academy of Agriculture and Forestry Sciences, Langfang, China; ^4^ State-owned Assets Supervision and Administration, Hebei Agricultural University, Baoding, China

**Keywords:** NAC transcription factors, woody plants, growth and development, adversity and coercion, regulatory mechanisms

## Abstract

The plant-specific NAC transcription factor family has the largest number of members, they are involved in regulating the entire process of plant growth and development, and the responses to biotic and abiotic stressors. This article reviews the progress regarding the elucidation of the structure and function of the NAC transcription factor family and the regulatory mechanisms through which NAC transcription factors affect various processes in woody plants, including the formation of the secondary cell wall, seed development, flowering, and fruit ripening. Additionally, this article encompasses the current research status of NAC transcription factors in response to abiotic stressors, such as salt and drought, as well as biotic stressors, such as pathogens and pests in woody plants. Most of the research is still at the stages of gene cloning, structural identification, and functional analysis; the specific downstream target genes and their molecular mechanisms remain unclear. Future research should focus on exploring unknown functions and action mechanisms, to promote an understanding of the regulatory network of NAC transcription factors in woody plants, thereby providing a theoretical basis and gene resources for research on NAC transcription factors and the creation of new forest germplasm.

Transcription factors (TFs), also called trans-acting factors, are DNA-binding protein molecules with sequence specificity that regulate gene expression. NAC TFs are among the largest TF family in plants (Duval et al., 2002). The name is derived from the initials of the *NAM* (no apical meristem) gene in *Petunia hybrida* (Souer et al., 1996), the *ATAF1/2* (*Arabidopsis* transcription activation factor) genes in *Arabidopsis thaliana* (Jensen et al., 2013), and the *CUC2* (cup-shaped cotyledon) gene in *A. thaliana* (Aida et al., 1997). The N-terminus of NAC TFs contains a highly conserved region of approximately 150 amino acid residues called the NAC domain, while the C-terminus is a diverse transcriptional regulatory region. NAC TFs have been found in dozens of plants, and their structure, expression characteristics, and functions reveal remarkable results (Zhao et al., 2020a). The NAC TF family genes are promising for plant breeding. Although many studies have reported on the functions of NAC TF genes, the functions of most members remain unclear, particularly in woody plants. Therefore, this review elaborates on the discovery and structural characteristics of NAC TFs, further discusses the gene classification and identification of NAC TFs in woody plants, and summarizes their regulatory roles in various growth and development processes, including seed germination, organ senescence, secondary-wall formation, flowering, fruit ripening, regulation of plant height, root development, and hormonal regulation in woody plants. This article also elaborates on the key roles of NAC TFs in the responses of woody plants to biotic and abiotic stressors.

## 1 Discovery and structural features of NAC transcription factors

In 1996, researchers first isolated (Souer et al., 1996) the NAC TF NAM from *Petunia hybrida* and reported an association with embryonic development (Zhong et al., 2023). Subsequently, researchers successively identified a variety of NAC-type TFs from woody plants, such as *Eucommia ulmoides* (Zhang et al., 2023b) *Camellia nitidissima* (Liu et al., 2025), and *Salix matsudana* (Qian et al., 2024). Many NAC TF genes have been discovered and cloned. The plant TF database has 19,997 NAC TFs documented from 150 species (Liu, 2023).

**Figure 1:**
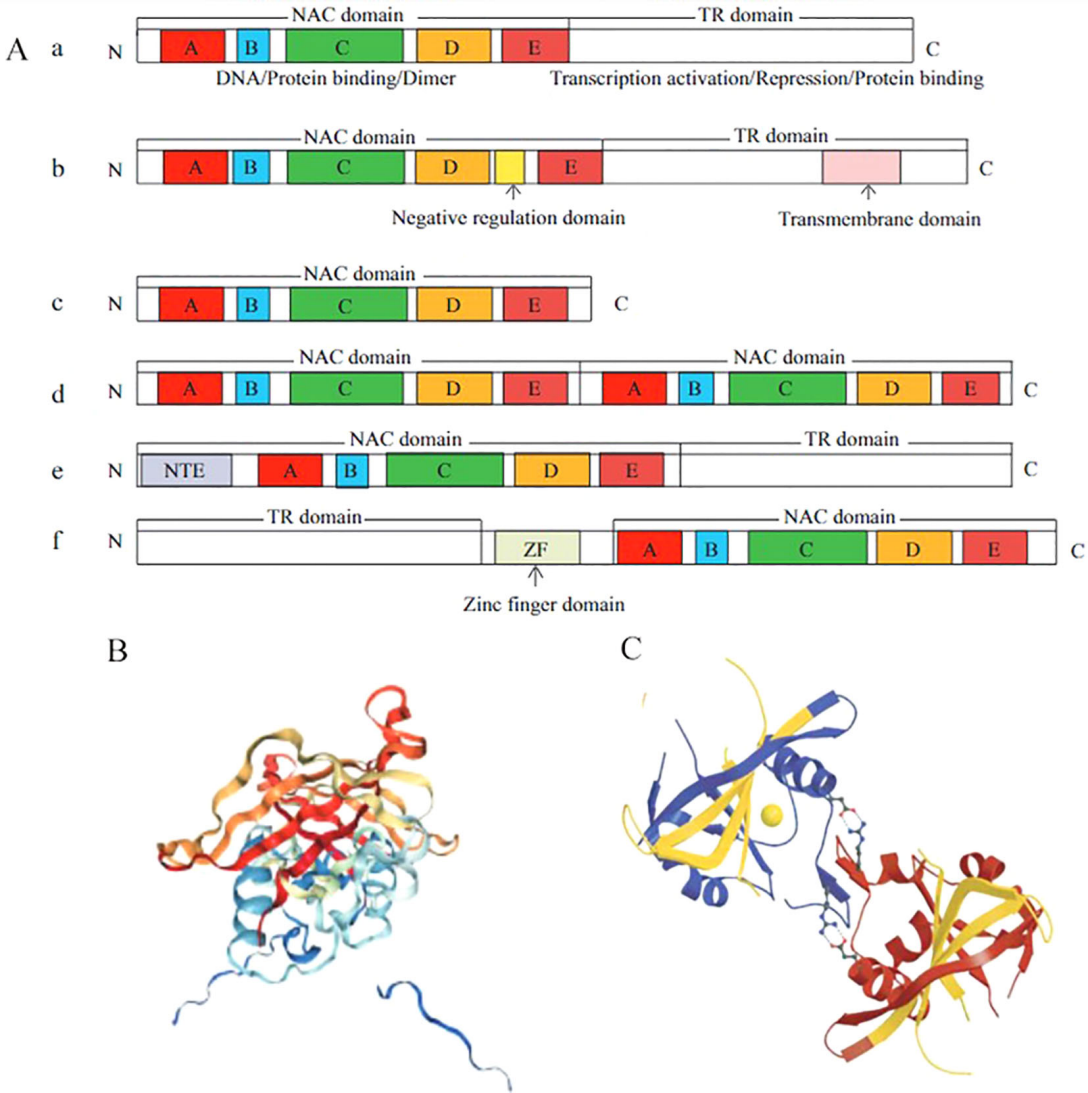
Structural characteristics of NAC transcription factors (Zhao et al., 2020a). Structural characteristics of NAC proteins **(A)**, TR: a highly variable transcription regulation **(A)**; Three-dimensional monomer structure of ANAC019 protein **(B)**; Homodimer structure of ANAC019 protein **(C)**.

The common feature of these TFs is the presence of the NAC domain at the N-terminus, which consists of 150 highly conserved amino acid residues. NAC TFs possess unique structural characteristics. A typical NAC TF consists of a conserved N-terminal protein domain and a variable C-terminal transcriptional regulatory region. The N terminus is involved in the specific binding of cis-acting elements, while the C terminus is responsible for regulating transcriptional activation (Ooka et al., 2003) as shown in **Figure 1A** (a). In contrast, as depicted in **Figure 1A** (b–f). An atypical NAC TF may contain a single NAC domain, two tandemly repeated NAC domains, or have the NAC domain at the C-terminus and a highly variable transcription regulation (TR) region at the N-terminus (Zhao et al., 2020a).The NAC domain is relatively complex, consisting of five subdomains (A, B, C, D, and E), while the NAM domain is composed of subdomains A, B, C, and D, among which subdomains A, C, and D are highly conserved in different species, and the C and D subdomains contain nuclear localization signals. However, subdomains B and E are less conserved (Li et al., 2018). ANAC019 from *Arabidopsis thaliana* is the first NAC protein to have its crystal structure determined. X-ray analysis revealed that the NAC domain is not a classical helix-turn-helix structure but a reverse parallel β-fold structure surrounded by several helices (Ernst et al., 2004) (**Figure 1B**), which form a conservative functional protein dimer through hydrogen bonding or a salt bridge between arginine (Arg) and glutamic (Glu) acid. In addition, many NAC proteins have dimer binding sites similar to ANAC019, and most NAC proteins bind to DNA in the form of homodimers, thus playing a regulatory role (Zhao et al., 2020a) (**Figure 1C**). The C-terminus of NAC TFs is a highly diverse transcriptional regulatory region, but some simple amino acids are frequently repeated. This region is rich in serine, threonine, proline, and glutamate, which leads to the loss of intrinsic disorder and a stable three-dimensional structure (Olsen et al., 2005).

Among the many NAC TFs, only a few have clear functions. Research on most NAC TF members is still at the level of gene cloning, structural identification, and expression analysis, and the downstream target genes and upstream regulatory factors of NAC TFs are poorly understood (Xu, 2008).

## 2 Classification and identification of NAC transcription factors

**Figure 2:**
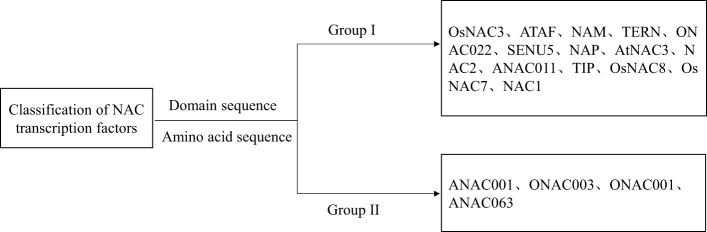
Classification of NAC transcription factors.

The NAC TF family has been extensively studied in the model plants *A. thaliana* and rice, and its classification is rather complex (Huang et al., 2017). The most commonly used method is to classify based on the characteristics of the NAC domain and a phylogenetic tree analysis. According to the similarity of the NAC domain and the protein amino acid sequences between *Oryza sativa* and *A. thaliana*, Ooka et al. (2003) divided the gene family into 2 categories (group I and group II) and 18 subgroups (group I contains 14 subgroups and group II contains 4 subgroups) (**Figure 2**). All subsequent classifications of NAC-TF subfamilies are primarily based on this study (Lin et al., 2023). For example, Rushton et al. (2008) analyzed the phylogeny of NAC TF family members in Arabidopsis, tobacco, rice, and other plants, and divided them into seven subfamilies, six of which exist in tobacco and other plants, and the seventh subfamily is unique to Solanaceae. Fang et al. (2008) conducted a phylogenetic tree analysis of NAC TFs in rice based on the coding amino acid sequences and divided them into groups I to V. Group I is related to plant growth and development and is further divided into 5 subgroups: I-1 (*OsNAC7*), I-2 (*NAC1*), I-3 (*NAM/CUC*), I-4 (*GRAB2*), and I-5 (*NAC2*). The tree structure of Group II is more complex than that of Group I, and it does not contain the published NAC sequences. Group III contains *NAC* genes related to stress (such as *ANAC019*, *ANAC055*, and *ANAC072* in *A. thaliana*), so it is named stress-related NAC (SNAC). Group IV contains 14 NAC members, and Group V has two NAC members. Most scholars use this method as the classification standard for the *NAC* gene family in woody plants.

For example, Lu et al. (2018) cloned and identified the *SNAC* genes *PeNAC034*, *PeNAC045*, and *PeNAC036* from *Populus euphratica*. Among them, *PeNAC034* and *PeNAC045* belong to the ATAF subgroup, while *PeNAC036* belongs to the *ANAC072* subgroup. Shang (2023) identified 21 *LiNAC* genes within the *Lagerstroemia indica* transcriptome data. The phylogenetic tree classified the *NAC* genes into two groups and 14 subgroups. The *LiNACs* genes in the subgroups exhibited similar conserved domains, gene structures, and analogous biological functions. Using the *A. thaliana NAC* genes as a reference, Zhang et al. (2023d) screened 76 NAC members and classified them into 17 subfamilies in *Cerasus humilis*, mainly including *OsNAC7, ANAC001, ONAC003*and other subfamilies, with the number of *ChNAC* family members in each subfamily varying and their functions potentially differing (Zhang et al., 2023d). Qi (2012) conducted a systematic analysis of the NAC family in *Populus*. A total of 163 *NAC* gene family members were identified. Phylogenetic analysis showed that these genes were clustered into 18 sub-families.

Many NAC TFs have been discovered through research on the woody plant genome. Researchers have identified different numbers of NAC TFs in woody plants (**Table 1**), and have explored the functions and mechanisms of the NAC TFs in the growth, development, and stress responses of woody plants. For example, Kang et al. (2021) identified 121 members in the NAC family within the entire *Juglans regia* genome. Among them, genes such as *JrNAC2*, *JrNAC8*, *JrNAC115*, and *JrNAC97*, may be key genes in the development of *Juglans regia* floral organs. Wang et al. (2024f) utilized bioinformatics methods to identify 92 members of the *NAC* gene family from *Morus alba*. They speculated that genes such as *MnNAC75*, *MnNAC104* and *MnNAC7*, play roles in resisting drought stress. Meng et al. (2022) identified 170 *NAC* genes from *Populus trichocarpa*, cloned 10 *PdNAC* genes from *Populus × euramericana* ‘Nanlin 895’, and analyzed their expression patterns under drought stress. Fu et al. (2024c) identified the *NAC* gene families of three *Castanea* species. Among them, *Castanea crenata Siebold* contains 118 *NAC* genes, *Castanea dentata* contains 100 *NAC* genes, and *Castanea mollissima* contains 115 *NAC* genes. They analyzed the evolutionary relationships, selection pressures, and codon preferences of the *NAC* gene family members in *Castanea* plants. Wang et al. (2024c) conducted a transcriptome analysis of different tissues in *Prunus persica* and screened out *PpNAC* genes that are specifically expressed in the fruit from 115 members of the *PpNAC* gene family, and analyzed the changes in their expression levels at different developmental stages. In addition, researchers have identified 62, 119, 51, and 73 NAC TFs from woody plants, such as *Pinus koraiensis Siebold*, *Osmanthus fragrans*, *Camellia gauchowensis*, and *Punica granatum*, respectively, and analyzed their roles in growth, development, and the stress responses (Kim et al., 2021; Yue et al., 2020; Shen et al., 2024; Li et al., 2021b).

**Table 1:** The number of NAC transcription factors in some woody plants.

Tree species	Gene number	References
*Juglans regia* L	121	Kang et al., 2021
*Morus alba* L	92	Wang et al., 2024f
*Populus trichocarpa* Torr. & Gray	170	Meng et al., 2022
*Populus × euramericanacv.* ‘Nanlin 895’	10	Meng et al., 2022
*Castanea crenata Siebold* et Zuccarini	118	Fu et al., 2024c
*Castanea dentata* (Michx.) Raf	100	Fu et al., 2024c
*Castanea mollissima* Blume	115	Fu et al., 2024c
*Amygdalus persica* Linn	115	Wang et al., 2024c
*Pinus koraiensis Siebold* et Zuccarini	62	Kim et al., 2021
*Osmanthus fragrans (Thunb.)* Loureiro	119	Yue et al., 2020
*Camellia gauchowensis* Chang	51	Shen et al., 2024
*Punica granatum* Linn	73	Li et al., 2021b

Most of the research on the functions of NAC TFs is concentrated on model plants, such as *A. thaliana*, *Nicotiana tabacum*, and *O. sativa*, while research on forest trees is lagging. Moreover, there are differences in the numbers, functions, and action mechanisms of NAC TFs among woody plants.

## 3 NAC transcription factors regulate the growth and development of woody plants

### 3.1 Participation in the formation of secondary walls

**Figure 3:**
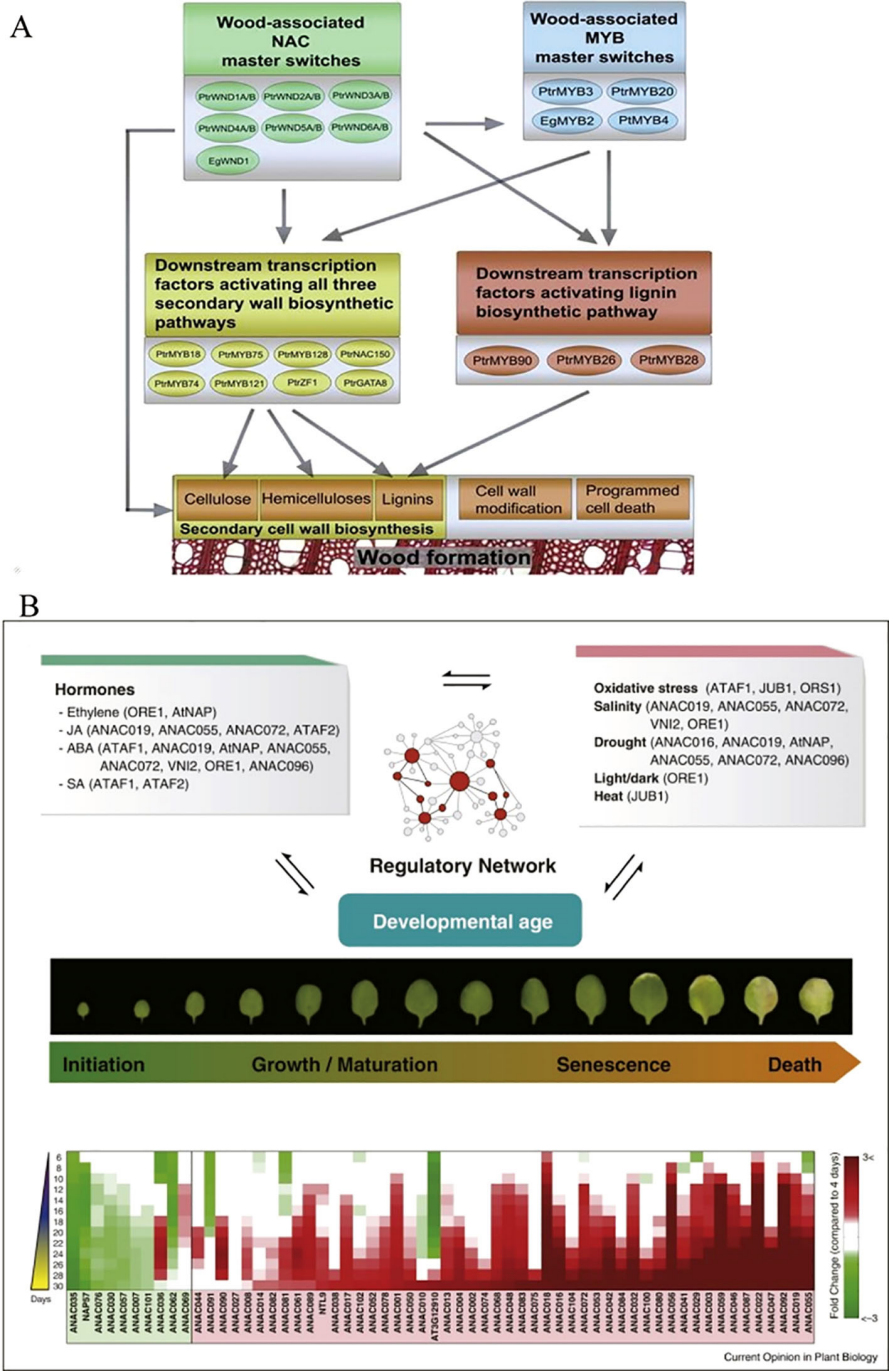
Role model of NAC in regulating secondary wall formation (Zhong et al., 2011) **(A)** and leaf senescence (Kim et al., 2016) **(B)**.

A group of TFs, including PtrNAC150, PtrNAC156, PtrNAC157, and PtrMYB18 have been revealed in poplar, a woody model plant (**Figure 3A**). These TFs activate the gene promoters of all three secondary wall biosynthetic pathways in the transient transactivation system using *Arabidopsis* protoplasts (Zhong et al., 2011) and regulate the gene expression of each component through a hierarchical NAC-MYB family transcription factor regulatory network (Hu, 2023). *BpNAC012* in *Betula platyphylla* is crucial for formation of the secondary wall. Once *BpNAC012* expression is inhibited, the deposition of the secondary wall in the stem fibers decreases significantly, and overexpression of *BpNAC012* activates the expression of downstream genes related to the secondary wall by directly binding to the site of the NAC binding element of the secondary wall, inducing ectopic deposition of the secondary wall in the stem epidermis (Hu et al., 2019). The NAC family member *SND2*, an indirect target of *SND1* and a key regulator of fiber secondary cell wall formation, plays a crucial role in the development of the secondary wall. Overexpression of *Arabidopsis SND2* increases the secondary wall thickness of fiber cells in *Eucalyptus*. This may be because eucalyptus trees have a stronger tolerance to high levels of *SND2* and/or the levels of *SND2* co-regulatory factors in the xylem, and the transcriptional level of *SND2* in eucalyptus trees remains moderate (Hussey et al., 2011). *SND2-B2* is a homolog of *Arabidopsis SND2* in poplar and promotes the synthesis of the secondary cell wall. When *SND2-B2* is overexpressed, the expression of genes related to synthesis of the secondary cell wall, such as the cellulose synthetic genes *PtrCesA17* and *PtrCesA18*, as well as the lignin biosynthetic genes *PtrCAld5H1* and *PtrCAld5H2*, are upregulated. The cell wall of secondary xylem fibers in transgenic plants becomes thicker; however, when the expression of *SND2-B2* is suppressed, the expression levels in transgenic plants are downregulated to varying degrees, in which the expression of xylan synthesis gene *PtrIRX9* was significantly down-regulated, and the secondary cell wall becomes thinner (Han, 2021). Alternative splicing of *PtrWND1B* in *Populus trichocarpa* occurs only in secondary xylem fiber cells. Overexpression of the normal short transcript of *PtrWND1B-S* in *Populus trichocarpa* enhances the thickening of the fibrous cell wall while overexpressing the *PtrWND1B-1* long transcript inhibits thickening of the fibrous cell wall (Zhao et al., 2014). In *Populus tomentosa*, genes encoding NAC domain proteins were isolated and named the *PtVNS* (*VND-, NST/SND-*, and *SMB-related protein*s) genes and the *PtrWND* genes includingVND and NST groups are driven under control of the cauliflower mosaic virus (CaMV) 35S promoter and induce thickening of the ectopic secondary walls in transgenic poplar leaves (Ohtani et al., 2011). The lignin content of the transgenic line overexpressing *PtrNAC128* in *P*. *trichocarpa* is significantly higher than that of the wild-type, which plays an important regulatory role in the synthesis of secondary cell wall components by activating the expression of key enzyme genes and TFs involved in lignin and cellulose biosynthesis (Li et al., 2020). The wood-related NAC domain protein 3 (PdWND3A) in *Populus deltoides* is a homologous sequence of VND4 and VND5 in *A. thaliana* and is involved in regulating synthesis of the secondary cell wall (Yang et al., 2019). The GUS activity driven by the *PtNAC068* promoter in transgenic *Populus tomentosa Carr* is present in the vascular tissues of stems, leaves, petioles, and roots. In contrast, the GUS activity driven by the PtNAC154 promoter only occurs in the secondary xylem of stems and leaf veins, indicating that upregulation of *PtNAC068* and *PtNAC154* is related to the secondary growth in poplars (Han et al., 2012). The NAC TF in *Pteroceltis tatarinowii* plays an important regulatory role in secondary growth (Zhou et al., 2025).

The secondary cell wall of plants is mainly composed of cellulose, lignin and hemicellulose. NAC TFs mainly participate in secondary cell wall formation by activating downstream TFs, increasing gene expression related to the synthesis of secondary cell wall components, such as cellulose and lignin, and regulating the biosynthesis of each component. Although some progress has been made in the biosynthesis of secondary wall components in model plants such as *Arabidopsis* and poplar, many pathways are unknown and need to be discussed in the fine regulatory framework of their component synthesis.

### 3.2 Participation in the regulation of organ aging

NAC TFs are important regulators of leaf senescence (Kim et al., 2016) (**Figure 3B**). The alternative *Populus* splicing variant *PtRD26IR* regulates leaf senescence by interacting with several central Sen-NAC TFs and inhibiting their DNA binding activities (Wang et al., 2021a). The heterologous expression of *DRL1* encoding a NAC TF in *Vitis vinifera* inhibits abscisic acid (ABA) synthesis, regulates the expression of related genes, and the content levels of 9-cis-epoxycarotenoid dioxygenase (NCED1), NCED5, zeaxanthin epoxidase 1 (ZEP1), ABA DEFICIENT2 (ABA2), ABA4, and ABA INSENSITIVE 2 (ABI2) decrease significantly, which reduces the sensitivity of plants to ABA, and delays leaf senescence in tobacco (Zhu et al., 2019). The *V. vinifera VviNAC33* gene is negatively regulated by miRNA164, which induces leaf chlorosis, inhibits organ growth, and directly activates the expression of STAY-GREEN protein 1, which is involved in the degradation of chlorophyll and the photosystems, and autophagy-related protein 8f (ATG8f), which participates in plant senescence (D’Incà et al., 2021). *CpNAC68* in *Chimonanthus praecox*, which is most highly expressed in old leaves and flowers, regulates physiological factors that provide stress tolerance during senescence (Lin et al., 2021). *Tectona grandis* TgNAC01 as a transcriptional activator of the ABA-mediated regulation and induces leaf senescence (Matias et al., 2022). In *Malus pumila*, MdNAC4 directly binds to the promoter of the senescence-associated gene *MdSAG39*, upregulating its expression, and positively regulates nitrogen deficiency-induced leaf senescence by enhancing ABA biosynthesis (Wen et al., 2023).

The leaves of woody plants are one of the most sensitive organs to senescence. Hormones, such as cytokinins, ethylene, jasmonic acid (JA), and salicylic acid, play important roles in organ senescence. NAC TFs play important roles in regulating secondary cell wall and organ senescence in woody plants, primarily by regulating the expression of related genes and plant hormone signal transduction pathways (ABA, ethylene, and JA). Most studies have focused on leaf senescence in annual plants, but the molecular mechanism of the regulation of leaf senescence in perennial woody plants remains unclear.

### 3.3 Participation in the regulation of seed development

**Figure 4:**
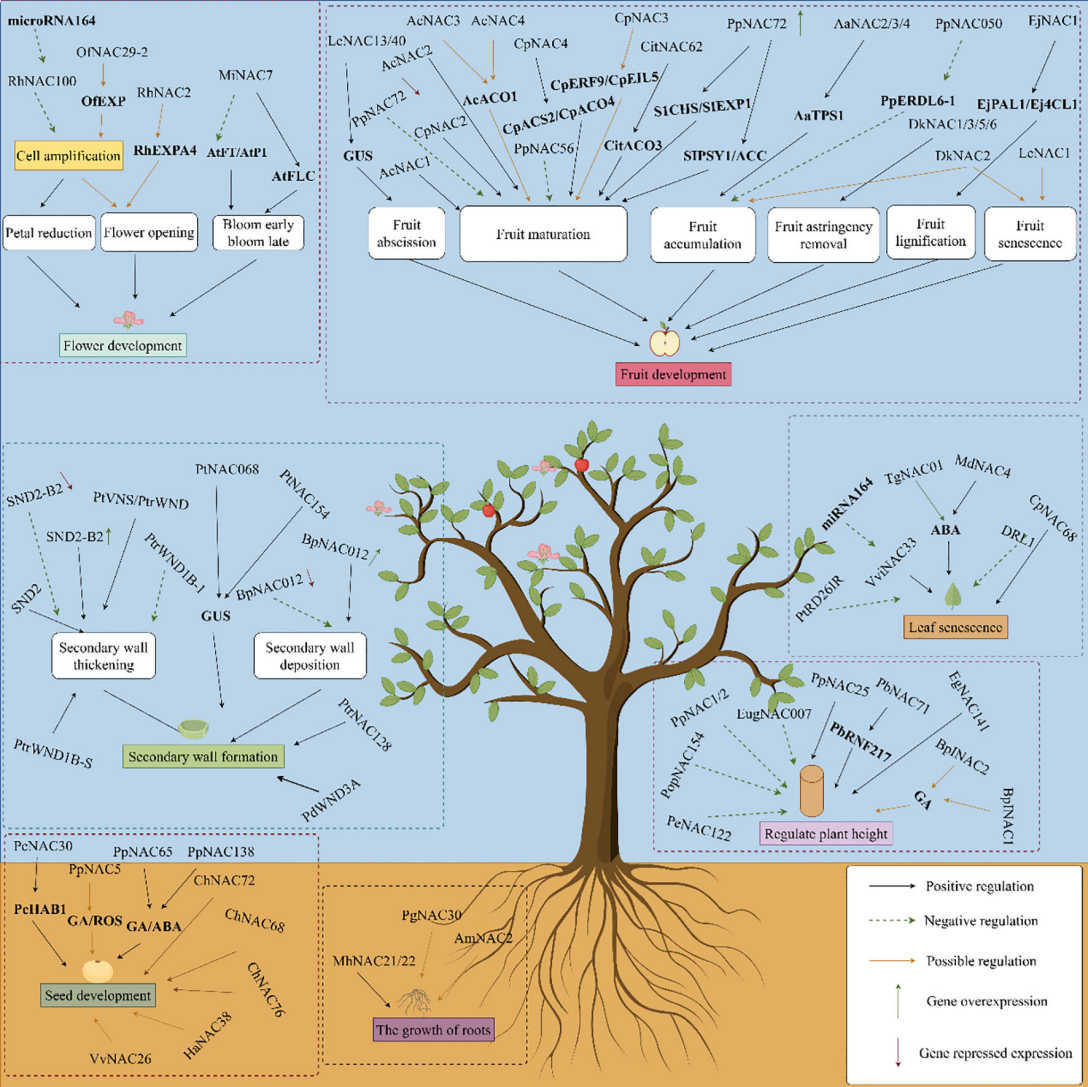
NAC transcription factors participate in the regulation of growth and development in woody plants (by Figdraw).

Perennial woody plants often have dormant seeds, and there is relatively little research into the specific molecular mechanisms. Seed dormancy and development are regulated by NAC TFs (**Figure 4**). The *PcNAC30* protein in *Pyrus calleryana* directly binds to the *PcHAB1* promoter, regulates expression of the *PcHAB1* gene, and controls release from seed dormancy (Bian, 2021). PpNAC5 may participate in the gibberellin (GA) and reactive oxygen species (ROS) synthetic pathways. PpNAC65 regulates the GA synthetic pathway, and PpNAC138 is involved in the GA and ABA synthetic pathways, thereby regulating the process of breaking seed dormancy in *P. calleryana* (Sun, 2019b). *VvNAC26* in *Vitis vinifera* may regulate seed development by affecting several hormone pathways and interacting with *VvMADS9* (Zhang et al., 2021a). In *Cerasus humilis*, genes such as *ChNAC68*, *ChNAC72*, and *ChNAC76* contain seed-specific cis-acting elements that are likely to play important roles in endosperm development (Zhang et al., 2023d). Under normal culture, *Arabidopsis* lines transfected with HaNAC38 from *Haloxylon ammodendron* exhibit significantly lower germination rates than wild-type lines, suggesting that NACs may delay or inhibit seed germination (Luo et al., 2023).

As plant-specific regulatory factors, NAC TFs play a key role in various processes by regulating the expression of related genes or interacting with other TFs, synthesizing hormones, and participating in hormone signal transduction pathways to induce and maintain seed dormancy and promote seed germination and other developmental processes.

### 3.4 Participation in the regulation of flowering

NAC TFs are also involved in the flowering of woody plants (**Figure 4**). The relevant *OfNAC* genes have been screened from the *Osmanthus fragrans* transcriptome. Among them, the *OfNAC29–2* gene may act like a regulator of *OfEXPA2* and *OfEXLA1* to control *OfEXP* and affect the expansion of petal cells. It has been speculated that *OfNAC* participates in the regulation of flower opening in *O. fragrans* (Miao et al., 2021). The *RhNAC2* gene in *Rosarugosa Thunb* may be involved in flower opening by regulating the expression of *RhEXPA4* (Dai et al., 2012). Ethylene regulates the accumulation of *RhNAC100* transcripts in *Rosarugosa Thunb* through microRNA164. Moreover, overexpression of *RhNAC100* in *Arabidopsis* inhibits the expansion of petal cells and negatively regulates their growth, leading to smaller petals (Pei et al., 2013). In *Mangifera indica*, *MiNAC7* affects the flowering of transgenic plants by regulating the expression of flowering-related genes (*AtFT, AtAP1*, and *AtFLC*) (Huang et al., 2023).

NAC TFs modulate the flowering process in woody plants by regulating the expression of related genes and adjusting post-transcriptional levels through miRNA164, thereby regulating hormone synthesis and flowering. However, the specific regulatory network needs further research.

### 3.5 Participation in the regulation of fruit development

NAC TFs play an important role in fruit growth and development (**Figure 4**). For example, promoters of the *LcNAC13/40* genes in *Litchi chinensis* drive the downstream *GUS* gene to be expressed in several organ abscission/shedding sites of *A. thaliana*, such as the abscission zones of floral organs, the abscission zones of cauline leaves, the seed abscission sites, and the pod dehiscent zones (Wen, 2019). Overexpression of *PpNAC72* in *Amygdalus persica* regulates the expression of a series of genes related to fruit development and maturation. The expression levels of the *S1CHS, SlEXP1, SlPSY1*, and *ACC* genes promote the growth and development of fruit, and speed up fruit maturation (Wu, 2021). When the *AdNAC3* gene from *Actinidia chinensis* is overexpressed in tomatoes, this gene promotes tomato fruit ripening, enhances the expression of genes related to ethylene synthesis, and accelerates the degradation of pectin (Yang et al., 2025). In *Citrus reticulata*, CitNAC62 and CitWRKY1 transactivate the *CitAco3* promoter, thus degrading citric acid during the development and ripening of citrus fruits (Li et al., 2017). Instantaneous silencing of the *PpNAC56* and *PpNAC72* genes in *A. persica* inhibits fruit ripening by reducing ethylene release and delaying fruit softening (Wang, 2024d). The TFs AcNAC1 and AcNAC2 in *Actinidia chinensis* participate in the ripening and softening processes by activating endo-transglucosylase/hydrolase genes (Fu et al., 2024a). Ethylene-induced *CpNAC4* mediates ethylene production by binding to and activating the promoters of ethylene biosynthetic-related genes *CpACS2* and *CpACO4*, thus participating in ripening of *Pseudocydonia sinensis* (Fu et al., 2023). A new MADS-box gene in *Pseudocydonia sinensis*, named CpMADS4, specifically binds and activates the promoters of ethylene signaling genes *CpERF9* and *CpEIL5*, together with *CpNAC3*, thereby possibly regulating fruit ripening (Fu et al., 2021). CpEIN3a interacts with CpNAC2 to individually or synergistically activate the transcription of gene subgroups related to carotenoid biosynthesis, thereby participating in the regulation of *Pseudocydonia sinensis* fruit ripening (Fu et al., 2017). Ethylene-induced AcNAC3 and AcNAC4 are transcriptional activators that may participate in ripening of kiwifruit by activating *AcACO1* (Fu et al., 2024b). *AaNAC2*, *AaNAC3*, and *AaNAC4* in *Actinidia arguta* bind to the terpene synthetase 1 promoter (*AaTPS1*), giving rise to the accumulation of more monoterpene volatiles in fruit (Nieuwenhuizen et al., 2015). The expression of *DkNAC1*, *DkNAC3*, *DkNAC5*, and *DkNAC6* is highly correlated with fruit astringency in persimmon, and *DkNAC2* may participate in processes related to fruit ripening or aging, but not in the removal of astringency (Min et al., 2015). *PpNAC050* increases fructose accumulation in flesh by inhibiting *PpERDL6–1* expression (Liu et al., 2024). EjNAC1 in *Eriobotrya japonica* regulates the expression of related genes (*EjPAL1* and *Ej4CL1*) and realizes fruit lignification (Xu et al., 2015). The expression of *LcNAC1* in *L. chinensis* fruit increases during the aging of the peel and flesh, which may promote fruit aging (Jiang et al., 2017).

As plant-specific regulatory factors, NAC TFs play key roles in a variety of processes. They regulate developmental processes, such as the induction and maintenance of seed dormancy and the promotion of seed germination, as well as a series of flowering and fruit development processes in woody plants. NAC TFs achieve this by regulating the expression of related genes, interacting with other TFs, and participating in hormone synthesis and hormone signal transduction pathways. However, the molecular mechanisms of how NAC TFs regulate the growth and development of woody plants are poorly understood, so further research is needed.

### 3.6 Regulation of plant height and root development

NAC TFs participate in the regulation of plant dwarfism traits (**Figure 4**). The *PpNAC1* and *PpNAC2* genes have been screened from *Prunus persica*. When the *PpNAC1* gene was transferred into poplar trees using the Agrobacterium-mediated method, stem thickness shrank, plant height decreased, and the leaves curled and wrinkled in the transformed *Populus* trees. Stem diameter and plant height also decrease in *Populus* trees transformed with the *PpNAC2* gene (Yang, 2022a). PbRNF217 in *Pyrus* promotes the ubiquitination and degradation of PbNAC71 dependent on the 26S proteasome, regulating the development of xylem and vessels and, thereby, affecting plant height (Cong, 2023). In *eucalyptus*, EgNAC141 is a positive regulatory factor in wood formation and participates in lignin synthesis (Sun et al., 2021). *EugNAC007* in *E. urophylla × E. grandis* is dominantly expressed in the xylem, while overexpression of this gene in poplar inhibits plant growth (He et al., 2023a). Overexpression of the *PpNAC25* gene increases the height of *Populus* trees, by increasing the number of internodes and promoting growth and development (Geng, 2023). Overexpression of the *PopNAC122* gene in *Arabidopsis*, a homolog of *XND1* in *Populus*, reduces the number of phloem fibers, xylem cell size and number, and vessel number while overexpressing the *PopNAC154* gene decreases plant height and increases the relative proportion of bark to xylem (Grant et al., 2010). In *Betula platyphylla*, BpINAC1 and BpINAC2 are induced by GA3, may participate in the GA signaling pathway, and regulate xylem development (Guo et al., 2015). When *PeNAC122* is overexpressed in *Populus euphratica*, plant height decreases, the xylem thickens, lignin accumulates, and the expression of genes related to secondary cell wall biosynthesis is upregulated (Chen et al., 2022).

Roots are one of the important organs for plant growth. Studies have shown that *NAC* genes affect the stress response in woody plants by regulating the growth and development of roots. The AmNAC2 protein in *Ammopiptanthus mongolicus* is highly expressed in the roots of wild plants and it may be involved in the growth and development of roots and leaves and related physiological activities (Zhang et al., 2021c). PgNAC30 in *Punica granatum* likely plays an important role in the development of the root system (Li et al., 2021b). The NAC TFs in *Casuarina equisetifolia* may be related to nitrogen fixation in root nodules, thereby participating in growth and development of plant roots (Xiao et al., 2025). Heterologous transformation of *A. thaliana* with *MhNAC21/22* was carried out with *Malus hupehensis*, and overexpressing *MhNAC21/22* promoted the formation of lateral roots (Cheng, 2024).

### 3.7 Regulation of hormonal balance

NAC TFs participate in a variety of hormone signal transduction processes. They exhibit complex regulatory functions during stress responses. NAC TFs also participate in the GA, ABA, ethylene, auxin, and cytokinin signal transduction pathways. For example, in *Carica papaya*, CpNAC2 directly binds to the *CpEIN3a* promoter, activates the transcription of a subgroup of genes related to carotenoid biosynthesis, such as *CpPDS2/4*, *CpZDS*, *CpLCY-e* and *CpCHY-b*, and regulates the synthesis of carotenoids during fruit ripening (Fu et al., 2017). *CpNAC4* is an ethylene-induced transcriptional activator that may mediate the production of ethylene by activating genes related to ethylene biosynthesis (Fu et al., 2023). *CpMADS4* (a novel MADS-box gene) and *CpNAC3* specifically bind to and activate the promoters of the ethylene signaling genes *CpERF9* and *CpEIL5*, thereby regulating the ethylene signaling transduction pathway during ripening of papaya fruit (Fu et al., 2021). The *CpNAC1* gene may promotes the synthesis of carotenoids during ripening in *Carica papaya* by regulating the expression of the *CpPDS2/4* gene (Fu et al., 2016). Ethylene-induced AcNAC3 and AcNAC4 in kiwifruit may participate in ripening and ethylene biosynthesis by activating *AcACO1* (Fu et al., 2024b). The *MdNAC42* gene promotes the synthesis of anthocyanins and proanthocyanidins in *Malus pumila* and regulates phenylalanine content in apples by controlling anthocyanidin reductase (Zhang et al., 2020b). *MdNAC18.1* indirectly regulates the transcriptional activities of key ethylene synthetic genes *MdACO1-like* and *MdACS1* by activating *MdNAC72* and *MdMYC2*. This forms a regulatory cascade that affects ethylene content (Zhang et al., 2025). The MdNAC52 transcript level increases during fruit coloring. Overexpression of *MdNAC52* promotes the accumulation of anthocyanins in the *Malus pumila* callus (Sun et al., 2019a). VcNAC072 interacts with the *AtPAP1* promoter and activates its expression, positively regulating the accumulation of anthocyanins in *Vaccinium vitis-idaea* fruit (Song et al., 2019). NAC6 in *Mangifera indica* may inhibit the expression of carotenoid cleavage dioxygenase, thus slowing the degradation rate of carotenoid components (Liang et al., 2024a). The *AsNAC22* gene in *Aquilaria sinensis* plays a role in the formation of secondary metabolites, such as chromones or sesquiterpenes (Yang et al., 2023). Heterologous expression of the *VaNAC26* gene from *Vitis amurensis* in *A. thaliana* upregulates the expression of JA synthesis genes and genes in the JA signaling pathway, thereby increasing JA content in *A. thaliana* (Fang et al., 2016).

NAC TFs mediate the production of hormones by activating the expression of relevant synthetic genes. They regulate the balance of hormones through multiple mechanisms and participate in their signal transduction pathways, playing an important role in growth, development, and stress responses.

## 4 NAC transcription factors are involved in the stress responses of woody plants

### 4.1 The salt stress response

NAC TFs respond to salt stress as shown in **Table 2**.

**Table 2:** The ways in which NAC transcription factors in some woody plants respond to salt stress.

Stress response mode	Gene	Species	Response results	References
Maintain the intracellular Na^+^ and K^+^ concentrations to enhance the salt tolerance of plants	*MdNAC17-L*	*Malus pumila* Mill	Regulate the distribution of Na^+^ by ion transporters to reduce the toxic effect of Na^+^	Su et al., 2022
	*PeNAC1*	*Populus euphratica* Oliv	Regulate Na^+^/K^+^ ion homeostasis to respond to salt stress.	Wang et al., 2013
	*Ptlinc-NAC72*	*Populus trichocarpa* Torr. & Gray	It participates in intracellular Na^+^ homeostasis, is induced by prolonged salt stress, and is mainly located in the cytoplasm.	Ye, 2021
Activate the expression of stress response-related genes	*ThNAC13*	*Tamarix hispida* Willd	Enhance ROS scavenging ability and regulate osmotic potential, thereby improving salt tolerance and permeability	Wang et al., 2017
	*ThNAC12*	*Tamarix chinensis* Lour	Enhance the removal ability of reactive oxygen species in plants, improve the ability of salt tolerance and osmotic stress tolerance of plants	Wang et al., 2021b
	*ThNAC7*	*Tamarix chinensis* Lour	It enhances the scavenging ability of reactive oxygen species in plants, thereby improving the tolerance of salt and osmotic stress of plants	He et al., 2019
	*CcNAC092*	*Cinnamomum camphora* (L.) Presl.	It contains abscisic acid response elements (ABRE), which enables *Cinnamomum camphora* to exhibit self-defense behaviors under salt stress.	Zeng et al., 2024
	*BpNAC2*	*Betula platyphylla* Suk	Promote the development of plant roots and enhance the ROS scavenging ability	Niu, 2022
	*AvNAC030*	*Actinidia chinensis Planch*	Improve the efficiency of active oxygen removal, balance the infiltration of outside and inside of cell, the integrity of the protective film, so as to improve plant salt tolerance	Li et al., 2021a
	*PtNAC101*	*Populus trichocarpa* Torr. & Gray	Inhibit the activity of antioxidant enzymes to reduce the antioxidant capacity of plants and negatively regulate the salt tolerance of plants.	Yang, 2021
	*MdNAC047*	*Malus pumila* Mill	It increases the expression of ethylene response genes, thereby enhancing the salt tolerance of *Maluspumila*	An et al., 2018b
	*PwNAC30*	*Picea wilsonii* Mast	It inhibits the tolerance of seedlings and mature plants to salt stress, and the accumulation of reactive oxygen species (ROS) increases significantly	Liang et al., 2020
	*PsnNAC090*	*Populus nigra* L	Enhanced ROS clearance in transgenic tobacco and reduced membrane lipid peroxide content to improve salt and osmotic tolerance	Wang et al., 2023
	*PeNAC034*	*Populus euphratica Oliv*	Downregulates the expression of several stress-related genes (such as COR47, RD29B, ERD11, RD22, and DREB2A), enhancing the salt stress sensitivity of transgenic plants	Lu et al., 2018
Promote the accumulation of small-molecule osmotic regulators	*NAC13*	*Populus* L	The increase in proline content enhances the salt tolerance of *Populus* trees	Zhang et al., 2019b
	*MbNAC29*	*Malus baccata*(L.)Borkh	The contents of chlorophyll and proline, as well as the antioxidant capacity, increase.	Han et al., 2020a
	*MbNAC25*	*Malus baccata*(L.)Borkh	The contents of proline and chlorophyll in the experimental plants have increased significantly compared with those in the control group, while the content of malondialdehyde has decreased	Han et al., 2020b
	*NAC57*	*Populus alba L*	It showed higher superoxide dismutase activity and peroxidase activity, and lower malondialdehyde content and relative conductivity than the wild type	Yao et al., 2018
	*PsnNAC054*	*Populus simonii×P.nigra*	SOD and POD activities, proline and chlorophyll contents were significantly higher than those of the wild type, and salt tolerance was enhanced	Wang, 2022b
Inducing stomatal closure	*VvNAC17*	*Vitis vinifera* L	The sensitivity to ABA is increased, and the ABA-induced stomatal closure is promoted	Ju et al., 2020
	*PeNAC045*	*Populus euphratica Oliv*	It significantly reduced the net photosynthetic rate, stomatal conductance and transpiration rate of poplar wild type (OEPeNAC045) under salt stress	Lu et al., 2018

**Figure 5:**
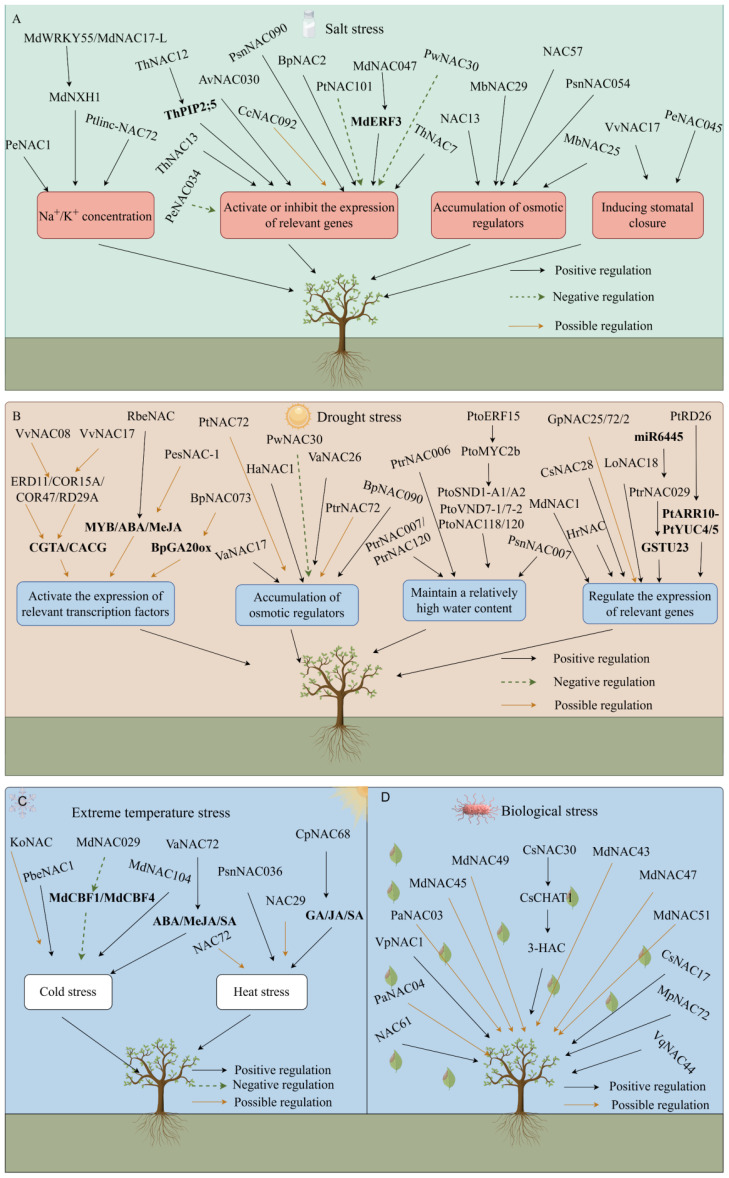
Interaction between *NAC* genes and various stresses (by Figdraw). Salt stress **(A)**; Drought stress **(B)**; Extreme temperature stress **(C)**; Biological stress **(D)**.

First, NAC TFs enhance salt tolerance in plants by maintaining the intracellular concentrations of Na^+^ and K^+^ (**Figure 5A**). When plants take in excessive amounts of Na^+^, NAC TFs distribute Na^+^ by regulating ion transporters. The protein complex formed by MdWRKY55 and MdNAC17-L in *Malus pumila* promotes the expression of the downstream *MdNHX1* gene, alleviating the toxic effects of Na^+^ (Su et al., 2022). The PeNAC1 TF in *Populus euphratica* responds to salt stress by regulating Na^+^/K^+^ ion homeostasis (Wang et al., 2013). Ptlinc-NAC72 in *Populus trichocarpa* is involved in intracellular Na^+^ homeostasis. It is induced by long-term salt stress and is mainly located in the cytoplasm (Ye, 2021).

NAC TFs maintain a relatively stable intracellular environment by activating the expression of stress response-related genes, such as *SOD, POD, ABA, RD22*, and ethylene (**Figure 5A**). Overexpression of *ThNAC13* TF in *Tamarix hispida* induces the activity of *superoxide dismutase* (*SOD*) and *peroxidase* (*POD*), improves salt tolerance and permeability by enhancing ROS scavenging ability and regulates osmotic potential (Wang et al., 2017). The ThNAC12 TFs directly regulate the expression of the *ThPIP2;5* genes, to scavenge ROS and improve salt tolerance (Wang et al., 2021b). Overexpression of *ThNAC7* enhances ROS scavenging, thereby improving tolerance to salt (He et al., 2019). *CcNAC092* contains an ABA response element, which prompts *Cinnamomum* camphora’s self-defense behavior under salt stress (Zeng et al., 2024). Overexpression of the *BpNAC2* gene promotes the development of roots and enhances ROS scavenging ability to improve salt tolerance in *Betula platyphylla* (Niu, 2022). In *Actinidia chinensis*, *AvNAC030* enhances salt tolerance by improving the removal efficiency of ROS, maintaining the osmotic balance inside and outside the cells, and protecting the integrity of the membrane (Li et al., 2021a). In *Populus trichocarpa*, *PtNAC101* inhibits antioxidant enzyme activities, reduces the antioxidant capacity of plants, and negatively regulates salt tolerance (Yang, 2021). MdNAC047 in *Malus pumila* directly binds to the *MdERF3* promoter (ethylene response factor) and activates its transcription, increasing the expression of ethylene-responsive genes, through a new regulatory pathway called MdNAC047-MdERF3-ethylene-salt tolerance, thereby enhancing salt stress tolerance in *Malus pumila* (An et al., 2018b). Transgenic *A. thaliana* with overexpressed *PwNAC30* from *Picea wilsonii* significantly inhibits salt stress tolerance in seedlings and mature plants, and ROS accumulation increases significantly (Liang et al., 2020). In *Populus nigra*, *PsnNAC090* improves salt and osmotic tolerance by enhancing ROS scavenging and reducing membrane lipid peroxide content in transgenic tobacco (Wang et al., 2023). In *Populus euphratica*, *PeNAC034* downregulates the expression of several stress-related genes (such as COR47, RD29B, ERD11, RD22, and DREB2A), thereby enhancing the salt stress sensitivity of transgenic plants (Lu et al., 2018).

NAC TFs promote the accumulation of small molecular osmotic regulators (such as betaine, proline, and soluble sugars) to protect the stability of cell structures (**Figure 5A**). Proline content increases in transgenic *Populus* trees overexpressing *NAC13* under salt stress, which enhances salt tolerance (Zhang et al., 2019b). Overexpressing *MbNAC29* from *Malus baccata* in *A. thaliana* results in higher chlorophyll and proline contents and antioxidant capacity under salt stress (Han et al., 2020a). When the *MbNAC25* gene of *M. baccata* is overexpressed in *A. thaliana* under high salt stress, proline and chlorophyll contents increase significantly compared with those of control plants. Additionally, malondialdehyde (MDA) content decreases, ROS species scavenging ability is enhanced, and the tolerance of plants to salt stress is promoted (Han et al., 2020b). Overexpression of *Populus alba NAC57* in *Arabidopsis* exhibits higher SOD and POD activity. The MDA content and relative electrical conductivity were lower than those of the wild-type, indicating that the *NAC57* gene plays an important role in the salt stress response (Yao et al., 2018). Under salt stress, plants overexpressing the *PsnNAC054* gene in *Populus simonii × P. nigra* have significantly higher SOD and POD activities, and higher proline and chlorophyll contents than wild-type plants, enhancing their salt tolerance (Wang, 2022b).

NAC TFs regulate stomatal closure to balance the water status within the plant (**Figure 5A**). The *heterologously* expressed *Vitis vinifera VvNAC17* gene increases the tolerance of *A. thaliana* to salt, osmotic, and freezing stress, increases ABA sensitivity, and promotes the ABA-induced stomatal closure (Ju et al., 2020). In *Populus euphratica*, Overexpression of *PeNAC045* leads to a significant decrease in the net photosynthetic rate, stomatal conductance, and transpiration rate of wild-type poplar (*OEPeNAC045*) under salt stress conditions (Lu et al., 2018).

**Figure 6:**
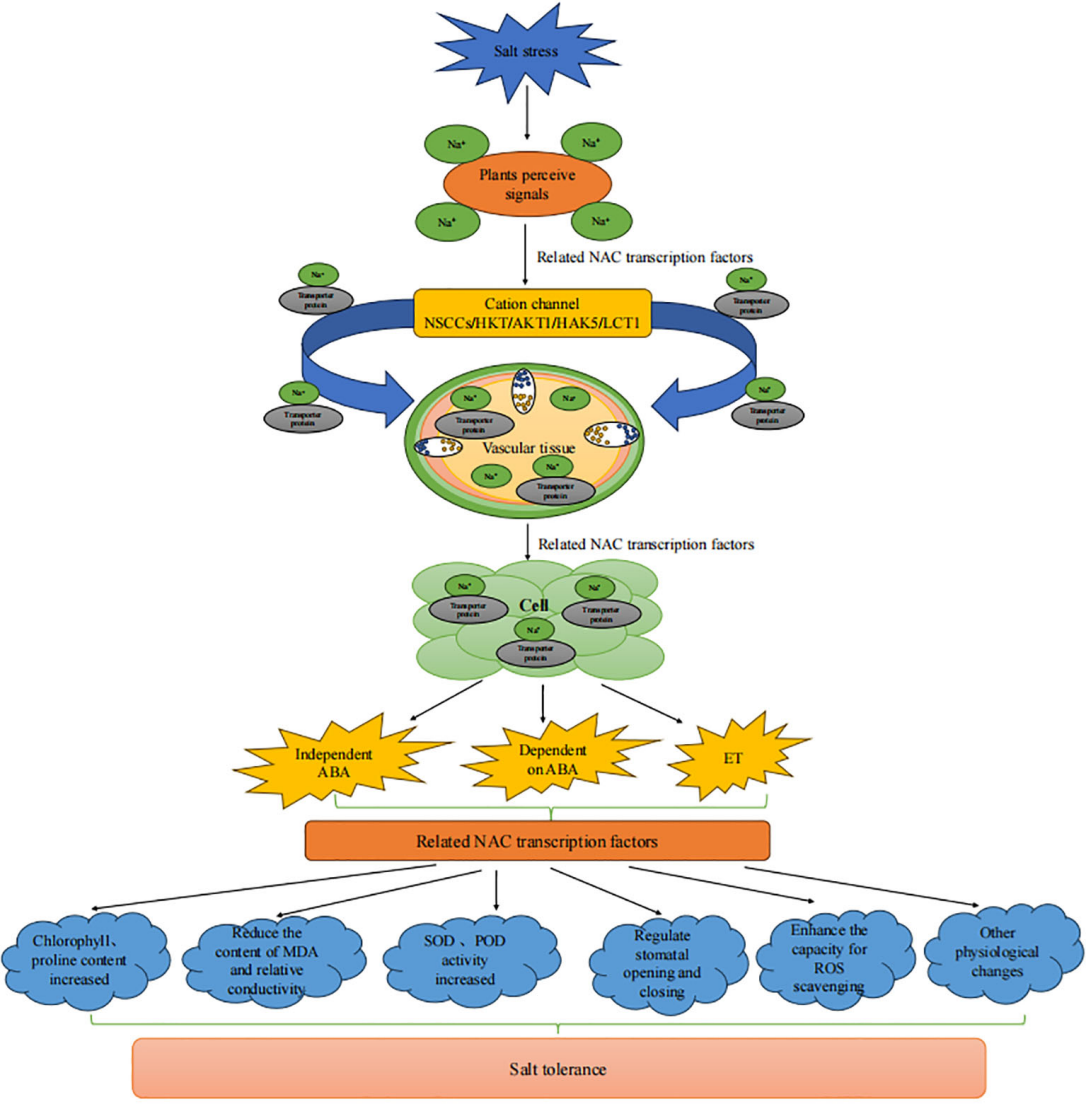
Response of NAC transcription factors to salt stress.

Under salt stress, external Na^+^ primarily enters the root cells via cation channels and transporters, such as nonselective cation channels (NSCCs), high-affinity K^+^ transporters (HKT), shaker-type potassium channels (AKT1), high-affinity K^+^ transporters (HAK5), low-affinity cation transporters (LCT1), etc (Kronzucker and Britto, 2011; Mian et al., 2011; Isayenkov and Maathuis, 2019). It then moves into plants through xylem loading and phloem recycling in stems (Van Zelm et al., 2020; Zhao et al., 2020b; **Figure 6**). NAC TFs simultaneously regulate multiple downstream stress-responsive genes and activate the salt tolerance response in plants. For example, NAC TFs respond to salt stress damage by regulating the transport of Na^+^ through cation transporters, activating the expression of stress response-related genes, mediating the ABA signaling pathway, accumulating osmotic regulators, balancing water status, and eliminating ROS through oxidative defense mechanisms to reduce oxidative damage.

### 4.2 The drought stress response

Plant NAC TFs improve plant drought tolerance through four pathways, as shown in **Table 3**.

**Table 3:** Ways in which NAC transcription factors in some woody plants respond to drought stress.

Stress response mode	Gene	Species	Response results	References
Integrate with cis-acting elements that respond to stress to activate the expression of relevant transcription factors	*VvNAC08 VvNAC17*	*Vitis vinifera* L	Enhance the tolerance of plants to drought stress	Ju, 2019
	*RbeNAC*	*Rosa berberifolia* Pall	The promoter contains a large number of MYB-binding sites, as well as ABA and MeJA elements	Feng et al., 2023
	*PeSNAC-1*	*Phyllostachys edulis* (Carrière) J. Houzeau	It may enhance the drought tolerance of transgenic rice by regulating genes in both ABA-dependent and ABA -independent signaling pathways	Hou et al., 2020.
	*BpNAC073* *BpDNMT1*	*Betula platyphylla*	The expression of BpGA20ox1 gene was affected by methylation to improve drought tolerance	Zhao, 2024
Increase the content of plant osmotic regulators	*PtNAC72*	*Citrus trifoliata* L	Negatively regulate the drought stress response of Citrus trifoliata	Xiong, 2020
	*HaNAC1*	*Haloxylon ammodendron* (C.A.Mey.) Bunge	The accumulation of proline, indoleacetic acid, and abscisic acid increases, and the drought tolerance of transgenic overexpressing lines is enhanced.	Gong et al., 2020
	*PtrNAC72*	*Citrus trifoliata* L	It negatively regulates the drought stress response by regulating the putrescine-related reactive oxygen species homeostasis	Wu et al., 2016a
	*BpNAC090*	*Betula platyphylla* Suk	Increase the content of hydrogen peroxide (H_2_O_2_) and the synthesis of proline (Pro) to reduce the damage caused by drought osmosis to *Betula platyphylla*	Wang, 2022c
	*VaNAC17*	*Vitis amurensis* Rupr	Enhance drought tolerance of transgenic Arabidopsis thaliana by regulating jasmonic acid biosynthesis	Su et al., 2020
	*VaNAC26*	*Vitis amurensis* Rupr	Up-regulated gene expression of jasmonic acid synthesis and jasmonic acid signaling pathway, increased jasmonic acid content, and enhanced its tolerance to drought	Fang et al., 2016
	*PwNAC30*	*Picea wilsonii Mast*	It reduces the drought tolerance of transgenic Arabidopsis thaliana by promoting the accumulation of reactive oxygen species (ROS) and inhibiting the expression of related genes	Liang, 2021
Maintain a relatively highwater content	*PtrNAC007* *PtrNAC120*	*Populus trichocarpa* Torr. & Gray	Enhance plant drought tolerance by changing the number and pore size of vessel cells	Li et al., 2019b
	*PtrNAC006*	*Populus×xiaohei* T.S. Hwanget Liang	Having stronger embolism tolerance enables plants to have enhanced drought tolerance	He, 2023b
	*PsnNAC007*	*Populus simonii×Pnigra*	Reduce transpiration and stomatal conductance to decrease water loss and increase the water use efficiency of plants	Wu et al., 2024
	*PtoERF15* *PtoMYC2b*	*Populus tomentosa* Carrière	It participates in the regulation of poplar duct morphology, maintains stem water potential and improves drought tolerance by regulating xylem duct development	Kong et al., 2023
Regulate the expression of relevant response genes	*MdNAC1*	*Malus pumila* Mill	The antioxidant capacity is enhanced, and the damage caused by drought stress is alleviated	Jia, 2019
	*CsNAC28*	*Camellia sinensis* (L.) Kuntze	Reduce the accumulation of reactive oxygen species (ROS) to improve dehydration tolerance	Zhang et al., 2023c
	*HrNAC*	*Hippophae rhamnoides* Linn	Activate the expression of drought-resistant related genes, thereby enhancing the drought tolerance of *Hippophae rhamnoides*	Zhang et al., 2023a
	*PtRD26*	*Populus* L	Induce the biosynthesis of cytokinins and the PtARR10-PtYUC4/5-mediated reactive oxygen species scavenging to improve the drought tolerance of *Populus*	Wang et al., 2022a
	*LoNAC18*	*Larix olgensis* Henry	The gene expression level is up-regulated, and the contents of SOD, POD, soluble proteins, and soluble sugars increase	Zhang et al., 2020a
	*PtrNAC029*	*Populus L*	Binding to the promoter of glutathione S-transferase GSTU23 and inhibiting its expression in response to drought stress in poplar	Niu et al., 2024
	*GpNAC25* *GpNAC72* *GpNAC2*	*Gymnocarpos przewalskii* Maxim	Under drought stress, gene expression was up-regulated in response to drought stress	Yang, 2024

NAC TFs bind to the cis-acting elements of key TFs responding to drought stress. The expression of related TFs is activated to improve plant drought tolerance, such as ABA, MYB, DREB, and WRKY (**Figure 5B**). The VvNAC08 and VvNAC17 TFs in *V. vinifera* may regulate the expression of these genes by binding to the core response elements ([CGTA/CACG]) of the promoter sequences of downstream target genes (*ERD11, COR15A, COR47*, and *RD29A*), thereby enhancing tolerance to drought stress (Ju, 2019). The *RbeNAC* promoter in *Rosa berberifolia* contains numerous MYB binding sites and ABA and methyl jasmonate elements are involved in drought-induced responses, indicating that they play an important role in drought stress (Feng et al., 2023). *PeSNAC-1* in *Phyllostachys edulis* may enhance drought tolerance in transgenic rice by regulating genes in the ABA-dependent and ABA-independent signaling pathways (Hou et al., 2020). In *Betula platyphylla*, *BpNAC073* and *BpDNMT1* proteins interact, potentially affecting *BpGA20ox1* expression via methylation to enhance drought tolerance (Zhao, 2024).

NAC TFs increase the contents of osmotic regulators, such as proline and betaine, within the plant, preventing protein denaturation and increasing the survival rate under drought stress (**Figure 5B**). The MDA content of *PtNAC72*-overexpressing *Citrus sinensis* plants is significantly higher than that of the wild-type, while the contents of proline, POD, and CAT are lower than those of the wild type, indicating that *PtNAC72* may negatively affects the drought stress response in *Poncirus trifoliata* (Xiong, 2020). Ectopic expression of *HaNAC1* in *Haloxylon ammodendron* leads to enhanced drought tolerance in transgenic overexpressing lines, the expression of stress-induced marker genes is upregulated, and the contents of proline, indole acetic acid, and ABA increase (Gong et al., 2020). *PtrNAC72* in trifoliate *Poncirus trifoliata* is a repressor of putrescine biosynthesis and may negatively regulates the drought stress response by modulating putrescine-related ROS homeostasis (Wu et al., 2016a). *BpNAC090* in *Betula platyphylla* reduces the contents of ROS and hydrogen peroxide (H__2__O__2__) and increases the synthesis of proline to mitigate the damage caused by drought-induced osmotic stress in *B. platyphylla* (Wang, 2022c). The TF VaNAC17 enhances drought tolerance of transgenic *A. thaliana* in *Vitis amurensis* by regulating JA synthesis (Su et al., 2020). *VaNAC26* upregulates genes involved in JA synthesis and signaling, increases JA content in *A. thaliana*, and enhances drought tolerance (Fang et al., 2016). *Picea wilsonii* PwNAC30 weakens the drought tolerance of transgenic *A. thaliana* by increasing the accumulation of ROS and inhibiting the expression of stress genes (Liang, 2021).

NAC TFs maintain a relatively high water content by inhibiting the outflow of water from cells (**Figure 5B**). *PtrNAC007* and *PtrNAC120* in *Populus trichocarpa* are highly induced by drought. Transgenic plants overexpressing these genes enhance their drought tolerance by altering the number and pore size of vessel cells (Li et al., 2019b). The soluble lignin and total lignin contents of *PtrNAC006* overexpressing plants increase significantly. After drought stress, the stomatal conductance of plants decreases, water loss decreases, water use efficiency increases, and the decline in hydraulic conductivity is mitigated, indicating that *PtrNAC006* overexpressing plants have stronger anti-embolism ability, thereby enhancing drought tolerance (He, 2023b). Transgenic *Populus simonii × P. nigra* plants overexpressing *PsnNAC007* reduce water loss by decreasing transpiration and stomatal conductance and increasing water use efficiency (Wu et al., 2024). Kong et al. (2023) identified *PtoERF15* and its target gene *PtoMYC2b* in *Populus tomentosa*. Three homologous gene groups in the NAC family, *PtoSND1-A1/A2*, *PtoVND7-1/7-2*, and *PtoNAC118/120*, are *PtoMYC2b* targets and regulate vessel morphology. They maintain the stem water potential by controlling development of xylem vessels, which enhances drought tolerance.

NAC TFs reduce the stress damage to plants by regulating the expression of drought stress-related response genes (**Figure 5B**). Overexpression of the *MdNAC1* gene in *Malus pumila* enhances antioxidant capacity under drought stress and alleviates the damage caused by drought stress (Jia, 2019). Overexpression of *CsNAC28* in *Camellia sinensis* confers hypersensitivity to ABA treatment in *Arabidopsis* and reduces the accumulation of ROS, thereby improving dehydration tolerance (Zhang et al., 2023c). Drought stress induces *HrNAC* in *Hippophae rhamnoides*, which activates the expression of drought-resistant related genes, thereby enhancing drought tolerance in sea buckthorn (Zhang et al., 2023a). The *PtRD26* promoter in *Populus* drives the expression of the isopentenyl transferase gene *IPT* to produce overexpressing plants. Transgenic plants improve drought tolerance in *Populus* by inducing the synthesis of cytokinin and PtARR10-PtYUC4/5-mediated ROS scavenging (Wang et al., 2022a). Expression of the *LoNAC18* gene in *Larix olgensis* is upregulated under drought stress conditions, and the contents of SOD, POD, soluble proteins, and soluble sugars increase, which participate in regulating the *L. olgensis* response to PEG-simulated drought stress (Zhang et al., 2020a). In poplar, *PtrNAC029* binds to the glutathione S-transferase *GSTU23* promoter and inhibits its expression. miR6445 directly targets *PtrNAC029*, and its upregulation downregulates *PtrNAC029*, leading to higher *GSTU23* expression. These three factors collectively respond to drought stress in poplar (Niu et al., 2024). Under drought stress, the expression levels of *GpNAC25*, *GpNAC72*, and *GpNAC2* in *Gymnocarpos przewalskii* are upregulated, indicating their potential roles in the drought response (Yang, 2024).

**Figure 7:**
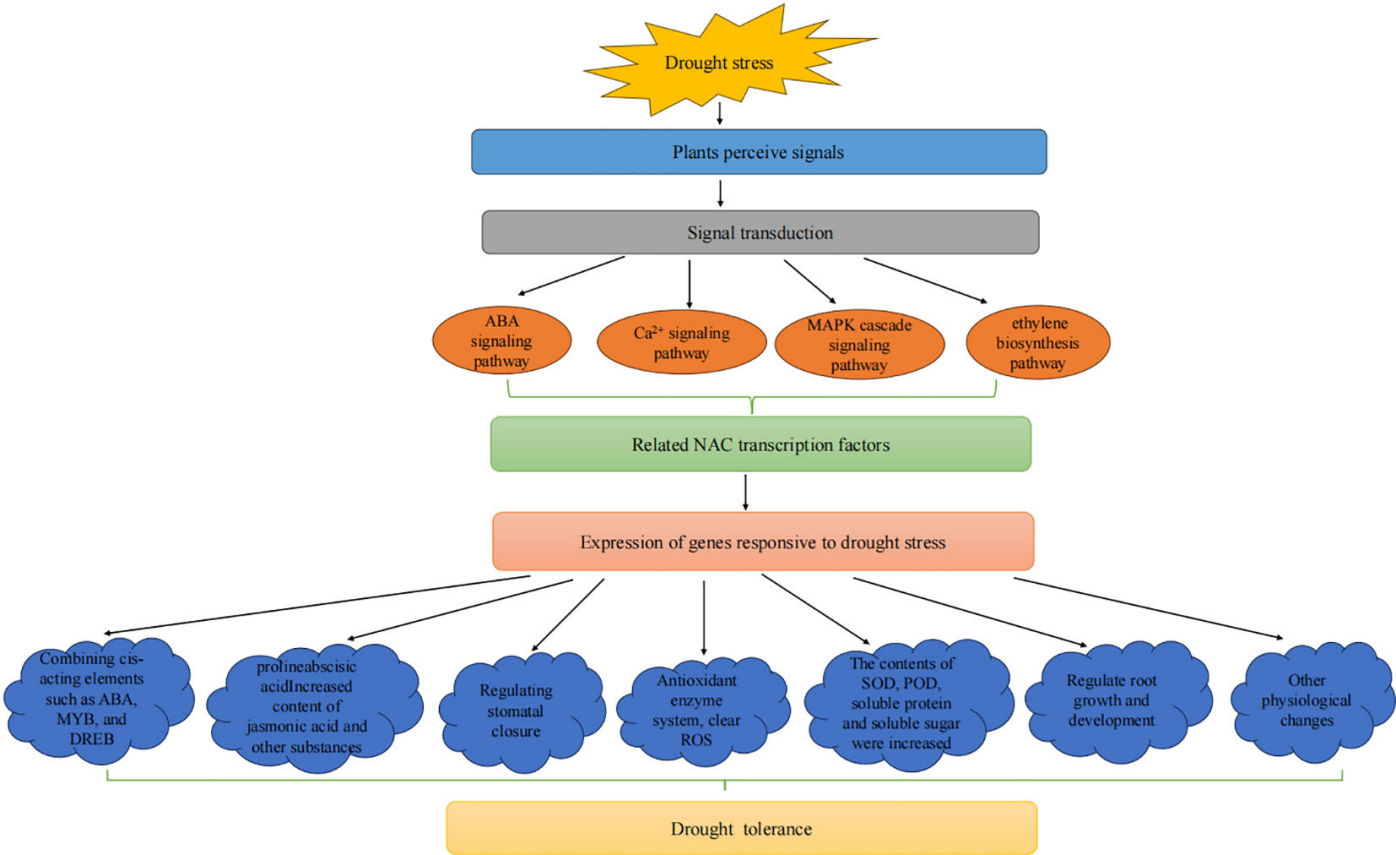
Response of NAC transcription factors to drought stress.

Plants mobilize various signaling pathways to respond to drought stress, including ABA, calcium ion, MAPKs cascade, and ethylene signaling pathways (Zhao, 2024; Liang et al., 2024b). NAC TFs also play essential roles in regulating these complex processes to respond to drought stress under varying environmental conditions (**Figure 7**). For example, ethylene affects osmotic regulatory substances and antioxidant enzyme systems, regulates ROS content, and participates in regulating stomatal opening and closing and inducing the expression of related genes by influencing ABA synthesis. In addition, *NAC* genes also affect the ability of plants to defend against stress by regulating the growth and development of roots. The *MiNAC1* gene in *Mangifera indica* improves the tolerance of transgenic plants to drought stress, salt stress, and low-temperature stress by increasing the length and number of lateral roots (Yang et al., 2022b). PwNAC11 has been isolated from *Picea wilsonii* and affects drought tolerance in transgenic *A. thaliana* by affecting root development (Zhang, 2019a).

### 4.3 The extreme temperature stress response

When plants are subjected to low-temperature stress, TFs activate the expression of low-temperature response genes by binding to cis-acting elements on gene promoters, thereby regulating the signal transduction pathways to improve low-temperature tolerance (**Figure 5C**). Overexpression of *PbeNAC1* in *Pyrus betulifolia* enhances tolerance to cold stress (Jin et al., 2017). Overexpression of *MdNAC029* in *Malus pumila* significantly inhibits the expression of *MdCBF1* and *MdCBF4* in the CBF pathway and, thus, the cold tolerance of plants is significantly downregulated (An et al., 2018a). Overexpression of *MdNAC104* affects the accumulation of osmotic regulatory substances in the stems and leaves of *M. pumila* under low-temperature freezing conditions, thereby enhancing cold tolerance (Mei, 2023). The *KoNAC* gene in *Kandelia obovata* contains a low-temperature response element LTR, which may play a role in the response to low-temperature stress (Sun et al., 2022). Under low-temperature stress, the expression of 10 NAC genes was significantly upregulated in plum blossoms, while the expression of five others was inhibited, indicating that the NAC TFs respond to low-temperature stress (Zhuo et al., 2018). In *Vitis amurensis*, VaNAC72, a TF regulated by the ABA, MeJA, and SA signaling pathways, positively regulates cold tolerance by acting upstream of *VaCP17* under cold stress (Qin et al., 2023).

Plants induce the expression of heat shock proteins under high-temperature conditions to maintain the stability of the cell membranes and remove excess ROS, thereby enhancing heat tolerance (**Figure 5C**). Overexpression of the *PsnNAC036* gene from *P. simonii × P. nigra* in tobacco promotes the growth of tobacco and enhances salt and heat tolerance (Zhang et al., 2021b). Woody plants also enhance their heat adaptive ability under various stressors through the ABA-dependent pathway. They close their stomata to reduce water transpiration and withstand heat stress. For example, *NAC29* and *NAC72* are upregulated under heat stress in *Rosa chinensis*, indicating that ABA signaling is likely to be involved in the heat stress response (Li et al., 2019c). The *CpNAC68* gene in *Chimonanthus praecox*, which activates nuclear protein transcription, positively regulates heat tolerance without affecting growth and development. It is induced by treatments, such as GA, JA, and SA (Lin et al., 2021).

NAC TFs have potential application value in improving the tolerance of woody plants to temperature stress. However, most research has focused on cold tolerance in woody plants. Only a few NAC TFs have been demonstrated to respond to high-temperature stress in woody plants, and the specific mechanisms of signal transduction and transcriptional regulatory networks require further study.

### 4.4 Participation in other abiotic stress responses

Woody plants are also subjected to other abiotic stressors in the environment. For example, *Salix integra* is a short-rotation woody plant with a high potential for phytoremediation of lead (Pb). *SiNAC* has a significant response to Pb, among them, the seven candidate transcripts of ATAF and NAP subfamilies, as well as the SiNAC137 transcript of SEIN5 subfamily, showed significant positive responses to lead stress and were significantly induced within 4 hours to 14 days after lead treatment. In addition, OsNAC7 and other subfamilies containing negative response transcripts may also participate in the lead response of integrated Streptomyces by reducing the activation of target genes (Xin et al., 2023). *MdNAC1* in *Malus domestica* is significantly induced by iron deficiency stress, enhances root acidification and promotes iron absorption by upregulating genes such as *AHA2/MDAHA12* and *FRO2* (Li et al., 2022). In Dangshan Su pears (*Pyrus bretschneideri*), the *PbrNAC* gene may respond to abiotic stressors, such as iron deficiency, by regulating root physiology and affecting chemical secretion and signal molecule transmission (Guo et al., 2023). *SmNAC007* in *Salix matsudana* may positively regulates responses to flooding stress by upregulating downstream genes involved in enzymatic detoxification and cellular protection, and increasing antioxidant proteins to reduce cell damage (Qian et al., 2024). Overexpressing *MhNAC21/22* in *Arabidopsis thaliana* from *Malus hupehensis* enhances root penetration ability, indicating positive regulation of root compression tolerance (Cheng, 2024). Under low nitrogen stress, the *NAC1* gene in *Betula luminifera* is regulated by miR164, and its expression rapidly increases to promote root development and enhance nitrogen absorption in seedlings (Wu et al., 2016b).

### 4.5 Participation in the biotic stress response

Plants are immobile, so they suffer adverse effects from the external environment during growth and development. Bacteria and fungi are significant biotic stressors (**Figure 5D**). For example, overexpression of *VpNAC1* in *Vitis vinifera* enhances the tolerance of transgenic tobacco to powdery mildew fungi and *Phytophthora nicotianae* (Zhu et al., 2012). The NAC TF NAC61 in grapevine regulates the *Botrytis cinerea*-susceptibility gene *WRKY52* (Foresti et al., 2023). VqNAC44 enhances stilbene synthesis and disease tolerance in Chinese wild grapes by interacting with VqMYB15 (Wang et al., 2024b). *PaNAC03* and *PaNAC04* may enhance tolerance to white rot infection in *Picea abies* (Dalman et al., 2017). The *NAC* genes *MdNAC43, MdNAC45, MdNAC47, MdNAC49*, and *MdNAC51* identified in *Malus pumila* are differentially expressed during infection with the *Alternaria alternata* apple pathotype, and their relative expression levels increase significantly, indicating that they may play a regulatory role in the tolerance of *M. pumila* to fungal diseases (Li et al., 2019a). Overexpressing *MpERF105* and *MpNAC72* boosts anthocyanin accumulation and enhances apple rust tolerance in leaves (Wang et al., 2024e). After *Ectropis obliqua* feeding, the tea plant (*Camellia sinensis*) activates JA signaling, upregulating *CsNAC30* and *CsTCP11*. This positively regulates *CsCHAT1* transcription, promotes (Z)-3-hexenyl acetate (3-HAC) synthesis in leaves, and enhances tolerance to *E. obliqua* (Gu et al., 2025). The interaction between CsNAC17 and CsbHLH62 activates CsRPM1, triggering the hypersensitive response and H_2_O_2_ accumulation, enhancing tea plant leaf tolerance to anthracnose (Han et al., 2024).

Under biotic stress, NAC TFs mainly participate in hormone-mediated disease resistance pathways, activating or repressing related gene expression to enhance plant adaptability and resistance. However, the specific mechanisms and pathways remains unknown.

## 5 Existing problems and prospects

NAC TFs have been discovered in dozens of plants. Remarkable achievements have been made in research on their structures, expression characteristics, and functions. They are a very promising family of genes for plant breeding. Through combined analysis of multiomics, such as genomics and proteomics, the structural features and characteristics of many forest tree NAC TFs have been identified, and it is more accurate to conduct functional verification research on forest tree NAC TFs with the development of gene editing technology. Many studies have reported on the gene functions of forest tree NAC TFs. However, most of the research has focused on whether *NAC* genes achieve the expected target effects in terms of growth, development, and stress responses after being transferred into plants, and their specific functions are still unclear. In particular, their roles and mechanisms in woody plants have not been fully elucidated. Most studies on the regulatory network and action mechanism of NAC TFs are concentrated at the levels of gene cloning, structural identification, and expression analysis, and there is a lack of in-depth research on specific downstream target genes and upstream regulatory factors of NAC TFs. Although previous studies have shown that NAC TFs play an important role in the growth, development, and stress responses of woody plants, their specific molecular mechanisms remain unclear, particularly in terms of the regulation of hormones and the signal transduction pathways. Therefore, in the future more forest tree NAC TFs will be mined through methods such as reverse genetics, genomics, and proteomics, and the functions of specific NAC TFs will be further explored through gene editing and transgenic technologies. It will be necessary to establish the regulatory network of NAC TFs, identify their downstream target genes and upstream regulatory factors, and combine multiomics data, such as the transcriptome, proteome, and metabolome, to analyze systematically the regulatory mechanisms and action pathways of NAC TFs, to understand more fully their roles in plant growth, development, and the stress responses, gradually improve the functional analysis of the NAC family in woody plants.

Cultivating superior forest tree varieties is currently the focus of forest tree genetic breeding research. The existing single superior traits are not meeting social needs, and comprehensive improvements in multiple target traits are required. Therefore, fully mining the key *NAC* genes from forest trees by combining existing superior forest tree varieties through genetic engineering methods and breeding forest tree varieties with high yield, strong adaptability, and stress tolerance, and applying them to production are needed. Most of the receptor plants for verifying *NAC* gene function are model plants, such as tobacco and *A. thaliana*. There are relatively more poplars among woody plants, while relatively fewer other plants are used in genetic transformation experiments, and there are even fewer reports on molecular breeding research in forest trees. Therefore, cross-species comparative studies are lacking, which prevents a comprehensive understanding of the functional differences in the NAC TFs in different woody plants. In recent years, genetic transformation and regeneration systems in common woody plants, such as poplar and pine, have progressed, but challenges remain. Most genetic transformations use Agrobacterium-mediated methods with antibiotics like kanamycin and hygromycin as markers, but this often results in false positives and chimeras, complicating screening and causing unstable gene expression. Transformation rates are low and cycles are long. While gene-editing technologies, such as CRISPR/Cas9, are widely used, their efficiency and stability need improvement and vary across species. In the future, this could be used as an entry point to mine more forest tree NAC TFs with high ecological and commercial value, conduct comparative studies of NAC TFs in different woody plants, explore their evolutionary processes and functional diversity, and establish an efficient, universal genetic transformation and regeneration system for co-transformation of multiple genes within a species or the same gene across species, improving the efficiency of forest tree traits. Additionally, clarifying the molecular mechanisms by which NAC TFs regulate the growth of forest trees and provide genetic resources, and establishing a theoretical basis for understanding their roles in plant adaptive evolution and forest tree trait improvement.

Most experiments on the regulation of growth, development, and stress responses in woody plants by NAC TFs use potted seedlings of common main cultivated varieties, so the conclusions apply to the seedling stage. However, woody plants are perennial with long growth cycles, and there are clear differences between the physiological functions and stress response mechanisms of seedlings and mature plants (Wu et al., 2015). As the plants grow, the types and content levels of secondary metabolites and hormones, as well as the functions of organs, such as roots, stems, leaves, flowers, and fruits, change accordingly. Similarly, it remains unclear whether the functions expressed by transgenic plants during the seedling stage also change later. Currently, the field trials of genetically modified forest trees mainly focus on the research of improving plant stress resistance. Whether there will be changes in their long-term physiological states and organ functions still requires more long-term field trials for monitoring. Therefore, in the future, the focus of experiments should not be limited to the seedling stage. Instead, transgenic plants should be planted for field trials. This will make it easier to observe whether the trait characteristics exhibited by transgenic plants under laboratory conditions can be maintained under long-term natural stress. Transgenic plants should be field-tested to observe if lab-demonstrated traits persist under long-term natural stress. This will allow for observations that cannot be seen during short-term stress at the seedling stage, such as damage to macromolecules (cell membranes, proteins, DNA) due to stomatal closure, impaired photosynthesis, and ROS accumulation, leading to a decline in physiological functions (Wei et al., 2020).

Hetero and homologous expression genes enable transgenic plants to obtain excellent tolerance and adapt more effectively to the environment. However, while the target genes are being expressed, they may interfere with the normal expression of host genes, leading to the upregulation or downregulation of genes, producing unexpected effects. For example, after some genes are transferred into plants, overexpression (strong constitutive promoters) leads to negative effects in the plants, as slow growth and organ senescence occur, harmful secondary metabolites accumulate, and adaptability of the stress response is weaker than expected (Shao et al., 2015). Some foreign *NAC* genes promote or inhibit stress tolerance without affecting growth. For example, *CpNAC68* in *Chimonanthus praecox* enhances heat tolerance without impacting growth (Lin et al., 2021). Conversely, some foreign genes affect the growth and development of plants while also enhancing stress tolerance, such as by regulating root growth in transgenic plants (Han et al., 2015; Zhang, 2019a). NAC TFs often regulate plant growth, development, and stress responses. Under natural conditions, most plants face multiple simultaneous stressors, such as drought, high temperature, and salinity (Shao et al., 2015). Under these conditions, enabling transgenic plants to cope with several stressors without hindering growth or even positively regulating it, to achieve expected benefits remains a challenge. The focus of future research should shift toward more in-depth research on the molecular mechanisms that produce unexpected effects, and whether it is possible to counteract some adverse effects by using weaker or specific promoters to drive gene overexpression. Moreover, as plants grow, they have some self-repair ability against negative impacts from the introduction of foreign genes. It remains unclear whether foreign gene expression and early-stage traits change with growth and environmental change. Therefore, we should continuously observe gene function and expression in adult trees to understand more fully the changing effects of foreign genes on recipient plants and obtain high-quality trees with excellent genetic traits.

Currently, research on NAC TFs in woody plants has focused on growth, development, and stress responses, with less attention on ecological adaptability. Introducing a foreign gene into a recipient plant could impact nearby genes, affecting certain plant traits. Within the gene flow range of genetically modified plants, if there are weed species that are genetically compatible for hybridization, field planting of transgenic plants may cause gene flow, leading to mutations in surrounding plants and weed overgrowth. Large-scale cultivation would reduce the number of traditional plants, disrupting ecological balance (Wang et al., 2024a; Huang, 2022). Therefore, long-term ecological monitoring is needed in the later stage to assess its impact on the ecosystem. The adaptability of plants relies on the integration of various stress response pathways under a constantly changing environment (Xu et al., 2024, 2025). Domestic and foreign scholars have relatively few research reports on the extreme temperature stress response of woody plant NAC TFs. Due to limited research on the heat stress response, only a few *NAC* genes have been confirmed to respond to extreme temperatures in woody plants. Moreover, current research is mostly biased toward the regulatory role of *NAC* genes under a single high-temperature stress, and further research is required to elucidate their related signal transmission and transcriptional regulatory networks. Relying on gene editing technologies, such as CRISPR/Cas9, will allow us to identify and utilize extreme temperature stress-related NAC TFs, and study the role of woody plant NAC TFs in ecological adaptation, particularly their performance under climate change and other environmental pressures, thus providing a theoretical basis for the cultivation and protection of woody plants.

In conclusion, although great progress has been made in research on NAC TFs since 1996, many mysteries are yet to be solved. Solving these mysteries and conducting relevant research will help to deepen our understanding of NAC TFs in woody plants and provide important theoretical bases and gene resources for plant breeding and ecological protection. With the development of molecular biology techniques, and employing genetic engineering methods such as genetic transformation and utilizing the characteristics of the NAC TFs, novel biotechnological approaches will be developed to improve certain plant traits and obtain superior new varieties, thereby enhancing the stress tolerance and productivity of woody plants, promoting the creation of new forest germplasm and providing broad application prospects in plant breeding.
